# Alzheimer’s disease pathogenesis: standing at the crossroad of lipid metabolism and immune response

**DOI:** 10.1186/s13024-025-00857-6

**Published:** 2025-06-04

**Authors:** Zitong Wang, Ling Zhang, Chuan Qin

**Affiliations:** 1 NHC Key Laboratory of Human Disease Comparative Medicine, Institute of Laboratory Animal Sciences, Chinese Academy of Medical Sciences (CAMS)& Comparative Medicine Center, Peking Union Medical College (PUMC), Beijing, 100021 China; 2 Changping Laboratory, Chinese Academy of Medical Sciences & Peking Union Medical College, Beijing, 102206 China; 3 National Human Diseases Animal Model Resource Center, 100021 Beijing, China; 4 National Center of Technology Innovation for animal model, 100021 Beijing, China

**Keywords:** Alzheimer's disease, Immune response, Lipid metabolism, Immunometabolism, Molecular mechanism

## Abstract

Alzheimer’s disease (AD) is a neurodegenerative disorder characterized by macroscopic features such as cortical atrophy, narrowing of the gyri, widening of the sulci, and enlargement of the ventricles. At the cellular level, the pathological characteristics include the extracellular aggregation of β-amyloid (Aβ) forming senile plaques, and the intracellular accumulation of hyperphosphorylated tau proteins forming neurofibrillary tangles. AD leads to the progressive decline of cognitive, behavioral, and social abilities, with no effective treatment available currently. The pathophysiology of AD is complex, involving mechanisms such as immune dysregulation and lipid metabolism alterations. Immune cells, such as microglia, can identify and clear pathological aggregates like Aβ early in the disease. However, prolonged or excessive activation of immune cells may trigger chronic neuroinflammation, thereby accelerating neuronal damage and the progression of AD. Lipid metabolism plays a critical role in maintaining cell membrane structure and function, regulating the production and clearance of Aβ, and supplying energy to the brain. Disruptions in these processes are closely linked to the pathological progression of AD. The interaction between lipid metabolism and the immune system further exacerbates the disease progression of AD. In this review, we discuss the lipid metabolism and immune response in AD, summarize their intricate interactions, and highlight the complexity of the multifactorial pathogenic cascade, offering insights into new interventions targeting the immune-metabolic axis in AD.

## Introduction

Alzheimer’s Disease (AD) is a common neurodegenerative disease characterized by progressive cognitive dysfunction and behavioral impairment in the elderly, and is one of the most prevalent forms of dementia worldwide. The main clinical manifestations of AD are the continuous deterioration of cognitive and memory function, the progressive decline in daily living abilities, and a variety of neurological, psychiatric symptoms and behavioral disorders [1]. Worldwide, at least 50 million individuals suffer from dementia, and this number is projected to reach 152 million by 2050 [2]. Currently, patients with AD often require long-term treatment and care, imposing significant economic and psychological burdens on both families and society [3, 4]. The pathological hallmarks of AD include the abnormal deposition of β-amyloid (Aβ) in the brain, forming amyloid plaques, and the pathological post-translational modifications and aggregation of tau protein within neurons, leading to neurofibrillary tangles [5]. Several hypotheses have been proposed to explain the potential mechanisms underlying AD pathogenesis, including the cholinergic hypothesis, the β-amyloid hypothesis, the tau hypothesis, the genetic hypothesis, the immune-inflammatory hypothesis, and the oxidative stress hypothesis [6]. Each of these theories explores different aspects of AD pathology; however, the exact etiology of the disease remains unclear. In recent years, increasing attention has been given to the roles of immunity and lipid metabolism in AD, highlighting their potential contributions to disease progression.

Immunity refers to the ability of an organism to recognize and defend against pathogens, foreign substances, and harmful agents through a series of physiological and biochemical processes. It includes innate immunity, mediated by macrophages and other immune cells, and adaptive immunity, driven by T cells and B cells. This self-defense mechanism is crucial for maintaining homeostasis. In the context of AD, inflammation reflects an innate immune response primarily mediated by activated microglia and astrocytes, which respond to pathological stimuli such as Aβ accumulation and tau pathology. Multiple studies have reported that nonsteroidal anti-inflammatory drugs (NSAIDs) may have preventive and therapeutic effects against AD [7–9]. Increasing evidence also links changes in immune cell levels in dementia and AD, identifying spatial associations between infiltrating immune cells in the AD brain and neurons, microglia, Aβ, and tau pathology. In a recent study, Van Olst et al. [10] employed cytometry by time-of-flight (CyTOF) to comprehensively map peripheral immune changes in patients with mild cognitive impairment (MCI) or dementia due to AD. Their findings revealed significant adaptive immune signatures in AD patients, demonstrating a strong correlation between peripheral immune alterations and both early and late clinical stages of the disease.

Metabolism encompasses all biochemical reactions within cells, tissues, and organisms that sustain homeostasis. Broadly, cellular metabolism is defined by complex biochemical pathways involved in biomolecule synthesis, maintenance, and degradation [11]. A well-regulated metabolic system is essential for maintaining physiological functions such as growth, reproduction, structural integrity, and environmental responses [12]. The brain is highly enriched in lipids, and lipid metabolism dysregulation has been recognized as a key contributor to AD pathogenesis [13]. As early as 1907, Alois Alzheimer observed lipid granule accumulation in microglial cells, suggesting a potential link between lipid metabolism dysfunction and AD-related pathological changes. Clinically, lipidomics and metabolomics studies consistently indicate altered levels of various lipid species in the early stages of AD. Mechanistically, extensive research has uncovered multifaceted interactions between lipid metabolism and core AD pathophysiological processes, including amyloidogenesis, bioenergetic deficits, oxidative stress, neuroinflammation, and myelin degeneration [14].

Immunometabolism is an emerging research field that investigates the metabolic processes of immune cells and their influence on immune responses, elucidating the intricate interplay between metabolism and immunity [15]. The fundamental mechanism of immunometabolism involves cellular activation and antigen stimulation, which trigger metabolic reprogramming to support downstream signaling pathways. Typically, upon the transmission of stimulatory signals into the cell, downstream signaling events are initiated, leading to changes in cytokine secretion and other functionally relevant processes. On one hand, metabolic alterations influence immune cell activity, thereby modulating immune responses [16]. Different immune cell subtypes, depending on their developmental stage and functional demands, require distinct energy sources, requiring metabolic reprogramming for proper regulation [17]. On the other hand, the immune system can regulate systemic energy metabolism through inflammatory responses. Cytokines, as immune cell-derived signaling molecules, can activate metabolic pathways, promoting energy production and macromolecule synthesis. Investigating the interplay between the immune and metabolic systems could offer novel insights into the prevention and treatment of chronic diseases.

In recent years, accumulating evidence has highlighted the crucial roles of immunity and lipid metabolism in AD onset and progression. These two processes are intricately connected and mutually influence each other, collectively driving AD pathogenesis. Therefore, further exploration of the relationship between immunity, lipid metabolism, and AD is crucial for elucidating disease mechanisms and developing novel therapeutic strategies.

## Characteristics of the immune response in Alzheimer’s disease

### Overview of the brain’s immune system

The immune system is a complex network composed of various organs, cells, humoral factors, and cytokines, responsible for recognizing and eliminating invading pathogens and abnormal cells to maintain overall health [18]. Its function is broadly categorized into innate immunity and adaptive immunity. Innate immunity serves as the body’s first line of defense, comprising physical barriers (e.g., skin, mucosa), immune cells (e.g., macrophages, neutrophils, natural killer cells), and immune molecules (e.g., complement proteins, cytokines). This system responds rapidly to pathogens but lacks specificity. In contrast, adaptive immunity is highly specific and has immune memory, primarily mediated by T cells and B cells. T cells can be further divided into subtypes, such as helper T cells (Th cells) and cytotoxic T cells (Tc cells), which are activated upon recognizing antigenic peptides presented by antigen-presenting cells (APCs) via major histocompatibility complex (MHC) molecules. These activated T cells exert immunoregulatory or cytotoxic effects. B cells, on the other hand, produce antigen-specific antibodies that facilitate pathogen clearance through antibody-mediated immune responses [19].

The central nervous system (CNS), which consists of the brain and spinal cord, plays a crucial role in processing and transmitting information to the peripheral nervous system while regulating bodily functions. Within the CNS, a unique neuroimmune regulatory system exists [20]. In response to disease or injury, the CNS immune system mobilizes immune cells by releasing cytokines, prompting their differentiation into specialized immune cells based on the severity and location of the injury. These immune cells facilitate the recognition and clearance of inflammatory factors while aiding neuronal repair [21]. However, persistent immune cell activation may lead to further damage to the CNS [22, 23].

### Innate immunity in Alzheimer’s disease

Microglia are the primary immune cells of the CNS, originating from myeloid progenitor cells during embryonic development, and are widely distributed throughout the brain and spinal cord [24]. Under physiological conditions, microglia remain in a resting state, continuously extending and retracting their processes to monitor the surrounding environment and maintain neural homeostasis. They secrete neurotrophic factors such as brain-derived neurotrophic factor (BDNF) and nerve growth factor (NGF), which promote neuronal survival, growth, and synaptic plasticity [25]^,^ [26]. Additionally, microglia regulate neurovascular units, maintaining cerebral blood supply and molecular exchange [27].

In the early stages of AD, Aβ deposition triggers microglial activation, shifting them from a resting to an activated state [28]. This is characterized by increased cell body size, shortened processes, and enhanced phagocytic capacity, allowing microglia to clear pathogens, apoptotic cells, and neurotoxic substances [29]. However, activated microglia also release pro-inflammatory cytokines such as tumor necrosis factor-alpha (TNF-α), interleukin-1β (IL-1β), and interleukin-6 (IL-6), as well as chemokines that recruit peripheral immune cells into the CNS, thereby initiating neuroinflammatory responses [30, 31]. While neuroinflammation may initially contribute to pathogen clearance and tissue repair, chronic or excessive neuroinflammation exacerbates neuronal damage and promotes neurodegeneration. Moreover, overactivated microglia may undergo phenotypic shifts, transitioning from a neuroprotective M2 phenotype to a neurotoxic M1 phenotype, which exacerbates neuroinflammation and neurodegeneration [32, 33]. The NLRP3 inflammasome, an inflammation-sensing protein complex, is highly expressed in activated microglia and macrophages. Its activation leads to the formation of oligomeric complexes containing apoptosis-associated speck-like protein (ASC) and caspase-1, perpetuating neuroinflammation. Knockdown of NLRP3 enhances Aβ phagocytosis by microglia, reduces their pro-inflammatory response and pyroptosis, and improves spatial learning and memory in 5×FAD mice [34].

Astrocytes, another major type of glial cell in the CNS, play key roles in neuronal metabolic support and ion homeostasis [35]. In AD, Aβ deposition and tau hyperphosphorylation directly activate astrocytes [36]. Furthermore, cytokines secreted by activated microglia, such as IL-1α, TNF-α, and C1q, can transform astrocytes from a resting to an activated state [37]. Activated astrocytes release large amounts of pro-inflammatory cytokines and chemokines, such as CXCL10, CCL2, IL-6, and BAFF, which further recruit immune cells to affected regions, exacerbating inflammation [38]. Additionally, astrocytes can release excitatory neurotransmitters such as glutamate, leading to excessive neuronal excitation, calcium influx, and subsequent neuronal damage [39]. Thus, astrocyte overactivation is a key pathogenic mechanism in AD.

The complement system, a crucial component of innate immunity, is abnormally activated in the AD brain, promoting Aβ deposition and neuroinflammation. The classical and alternative complement pathways identify and tag Aβ, facilitating its phagocytosis by microglia [40]. However, complement system dysregulation may also cause neuronal damage, contributing to AD pathology [41, 42]. C1q, an initiator of the classical complement cascade, plays a critical role in synaptic toxicity induced by soluble Aβ oligomers. C1q deficiency reduces astrocyte-synapse interactions in tauopathy models and decreases microglial phagocytosis of synapses [43–45]. Additionally, in response to soluble Aβ oligomers, adult microglia phagocytose synapses in a CR3-dependent manner [46]. The C5b-9 membrane attack complex (MAC) can directly damage neuronal membranes, leading to neuronal death. Complement inhibition prevents MAC-mediated blood-brain barrier disruption in Tg-SwDI/B mice, demonstrating its potential therapeutic value [47].

### Adaptive immunity in Alzheimer’s disease

**Fig. 1:**
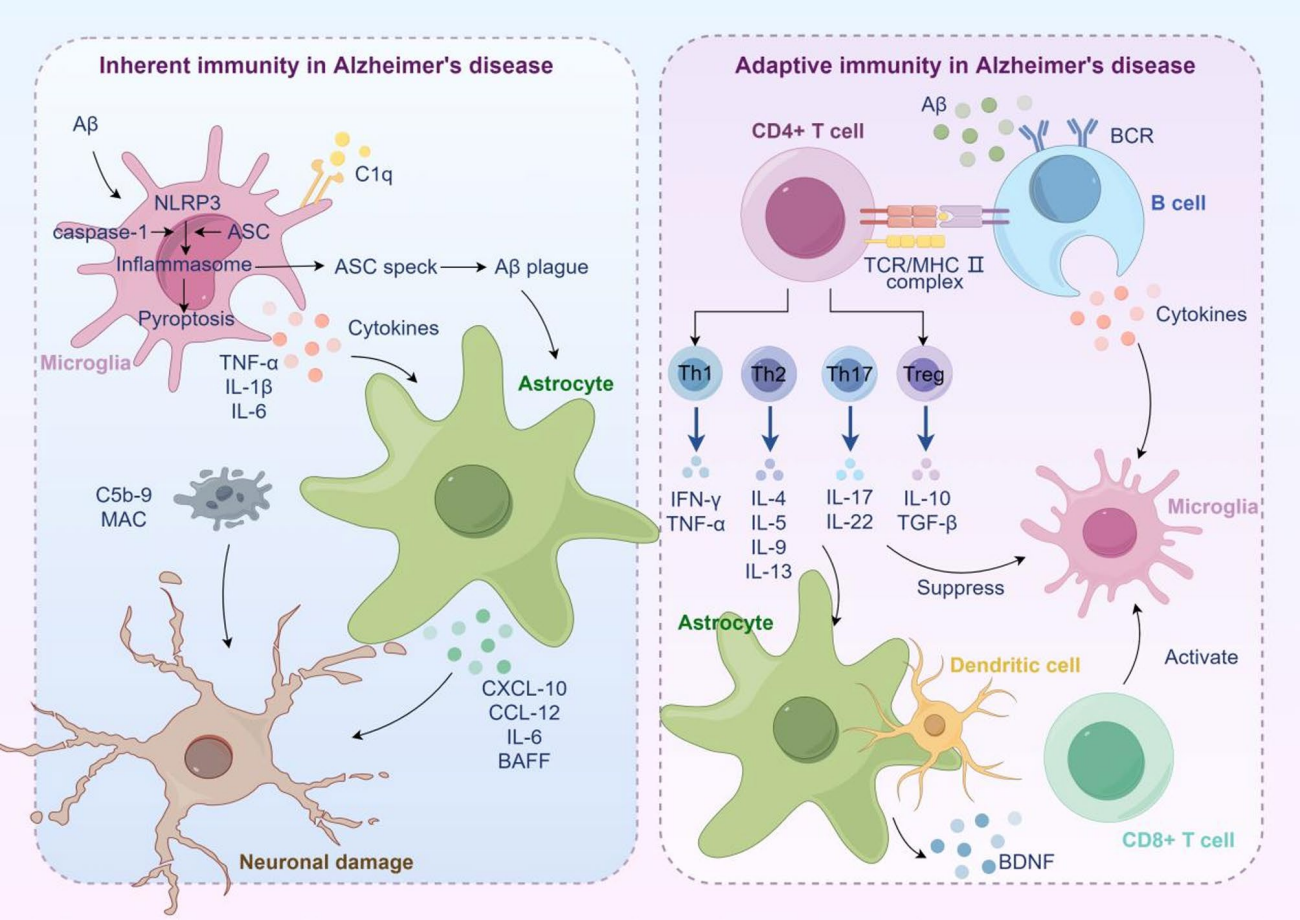
Immune response in Alzheimer’s Disease. Under the stimulation of Aβ, microglia are activated and secrete multiple inflammatory cytokines and chemokines, which trigger neuroinflammatory response. It also activates astrocytes, further exacerbating the inflammatory response. Activated T cells and B cells secrete pro-inflammatory and anti-inflammatory cytokines, regulate the state transition of microglia, as well as astrocytes and dendritic cells, and participate in the regulation of immunity and inflammation in the progression of AD. Overall, during the progression of AD, the immune response shifts from an activated and elevated state in the early stage, attempting to counteract pathological products such as Aβ, to a complex imbalanced state in the later stage. Excessive inflammatory reactions persist and mutually promote neurodegenerative changes. Meanwhile, although regulatory immune mechanisms are involved, they are difficult to restore immune balance

Recent evidence suggests the presence of adaptive immune responses in both the blood and cerebrospinal fluid (CSF) of AD patients [48, 49]. Peripheral immune cells—including neutrophils, T cells, B cells, natural killer (NK) cells, and monocytes—infiltrate the brain vasculature and parenchyma, modulating immune and inflammatory responses and playing critical roles in AD progression [50] (Fig. 1).

CD4 + T cells differentiate into subtypes such as Th1, Th2, Th17, and Treg cells. Under normal conditions, Th1 (producing IFN-γ) and Th2 (producing IL-4) cells reside in the meninges, where IFN-γ regulates meningeal dendritic cells and astrocytic BDNF expression, influencing neural circuits underlying social behavior. However, T-cell imbalance can lead to dysregulated immune-inflammatory responses. In AD, abnormal T-cell crosstalk with glial cells results in excessive pro-inflammatory cytokine secretion, promoting neuroinflammation and neurodegeneration [51, 52]. In tauopathy mouse models and AD brains, the number of T cells, especially cytotoxic T cells is significantly increased in tau pathology regions, correlating with neuronal loss [53]。.

The exact role of CD8 + T cells in AD remains elusive—both their depletion and accumulation can accelerate disease progression [54]. Studies have observed increased CD8 + T cell infiltration in the hippocampus of human AD and APP-PS1 mice [55]. Some studies have reported that brain CD8 + T cells limit AD pathology, including β -amyloid deposition and cognitive decline [56]. However, Jorfi et al. [57] observed the infiltration of CD8 + T cells into AD cultures resulted in increased microglial activation, neuroinflammation, and neurodegeneration by constructing a three-dimensional human neuroimmune axis model of stem cell-derived neurons, astrocytes, and microglia, and peripheral immune cells. Conversely, regulatory T cells (Tregs), particularly Aβ antigen-specific Tregs (Aβ + Tregs), suppress pro-inflammatory microglial activity, alleviating cognitive impairment, Aβ accumulation, tau hyperphosphorylation, and neuroinflammation in AD models [58]^,^ [59].

B cells, key components of adaptive immunity, also play complex roles in AD. They produce Aβ-targeting antibodies, present antigens to T cells, and regulate immune responses [60]. First, B cells can produce antibodies, and the induction of β amyloid plaque formation using 44B cells was used to construct AD cell models [61]. The increase in B cells is associated with increased brain amyloid deposition and with hyperactivation of induced pluripotent stem cell-derived microglia, leading to a loss of β -amyloid clearance function [62]. Loss of B cells also reduced Aβ plaque burden and disease-associated microglia, reversing behavioral and memory deficits and restoring TGF β + microglia, respectively, delaying AD progression in mice [63]. However, Feng et al. [64] reported that B cell-derived IL-35 was able to inhibit Aβ production in the frontal cortex in 5×FAD mice. Xiong et al. [65] detected a significant reduction in B cells in the blood of AD patients and identified 18 genes that were specifically up-or downregulated in B cells strongly associated with AD severity. Therefore, the exact contributions of B cells in the pathogenesis of AD require further investigation.

### Immune-related animal models of Alzheimer’s disease

In vivo models of AD aim to replicate the neuropathological features of the human disease in order to create platforms for studying how the disease develops and progresses, as well as for testing potential treatments that could modify its course. Using immune-related animal models, the investigators explore the role of inflammation in AD progression and aim to identify immune-modulating therapeutic targets. The following animal models have been developed over the years (Table 1).

**Table 1:** Immune-related animal models of Alzheimer’s disease

Model Type	Description	Intervention	Reference
Immunoinflammatory Animal Models	Focus on the roles of immunocytes and cytokines in AD	IL−1β overexpression in APPswe/PS−1dE9 mice	[66]
		IL−3 deficiency in 5xFAD mice	[67]
		IL−6 deficiency in 5xFAD mice	[31]
		IL−12 p40 deficiency in APP/PS1 mice	[68]
		IL−12 p40 deficiency in APP23 mice	[69]
		NLRP3-targeted 5×FAD mice	[34]
		CX3CR1 deficiency in 5xFAD mice	[70]
		CD33 deficiency in 5xFAD mice	[71]
		TREM2 deficiency in 5xFAD mice	[71]
		TREM2 overexpression on microglia in the brain of P301S mice	[72]
Animal Models with Induced Inflammation	Animals with induced inflammation to mimic neuroinflammation	lipopolysaccharide (LPS)-induced neuroinflammation in WT mice	[73, 74]
		LPS-induced neuroinflammation in 5xFAD mice	[75]
		LPS-induced neuroinflammation in APP/PS1 mice	[76, 77]
Animal Models of Immune Modulation Therapy	Immune-modulatory therapies to reduce neuroinflammation in AD models	IL−2 treatment for APP/PS1ΔE9 mice	[78]
		Low-dose IL−2 treatment for APP/PS1 mice	[79]
		anti-IL−17 treatment for 3xTg mice	[80]
		IL−17Ab treatment for CD−1 mice with Aβ injection	[81]
		IL−33 treatment for APP/PS1 mice	[82, 83]
		IL−35 injected in 5xFAD mice	[64]
		NSAIDs	[9]
		Minocycline (TNF-α Inhibitor) treatment for APP-tg mice	[84]
		Minocycline (TNF-α Inhibitor) treatment for APP/PS1 mice	[85]
		Imipramine (TNF-α Inhibitor) treatment for APP-tg mice	[86]
		Thalidomide (TNF-α Inhibitor) treatment for 3xTg mice	[87, 88]
		Etanercept (TNF-α Inhibitor) treatment for 3xTg mice	[89]
		TfRMAb-TNFR (TNF-α Inhibitor) treatment for APP/PS1 mice	[90]
		Complementary suppressor therapy	[47]
		Aβ-specific Tregs treatment in 3xTg mice	[59]
		TREM2 wild-type microglia transplantation therapy	[91]

## Characteristics of lipid metabolism in Alzheimer’s disease

### Overview of brain lipid metabolism

**Fig. 2:**
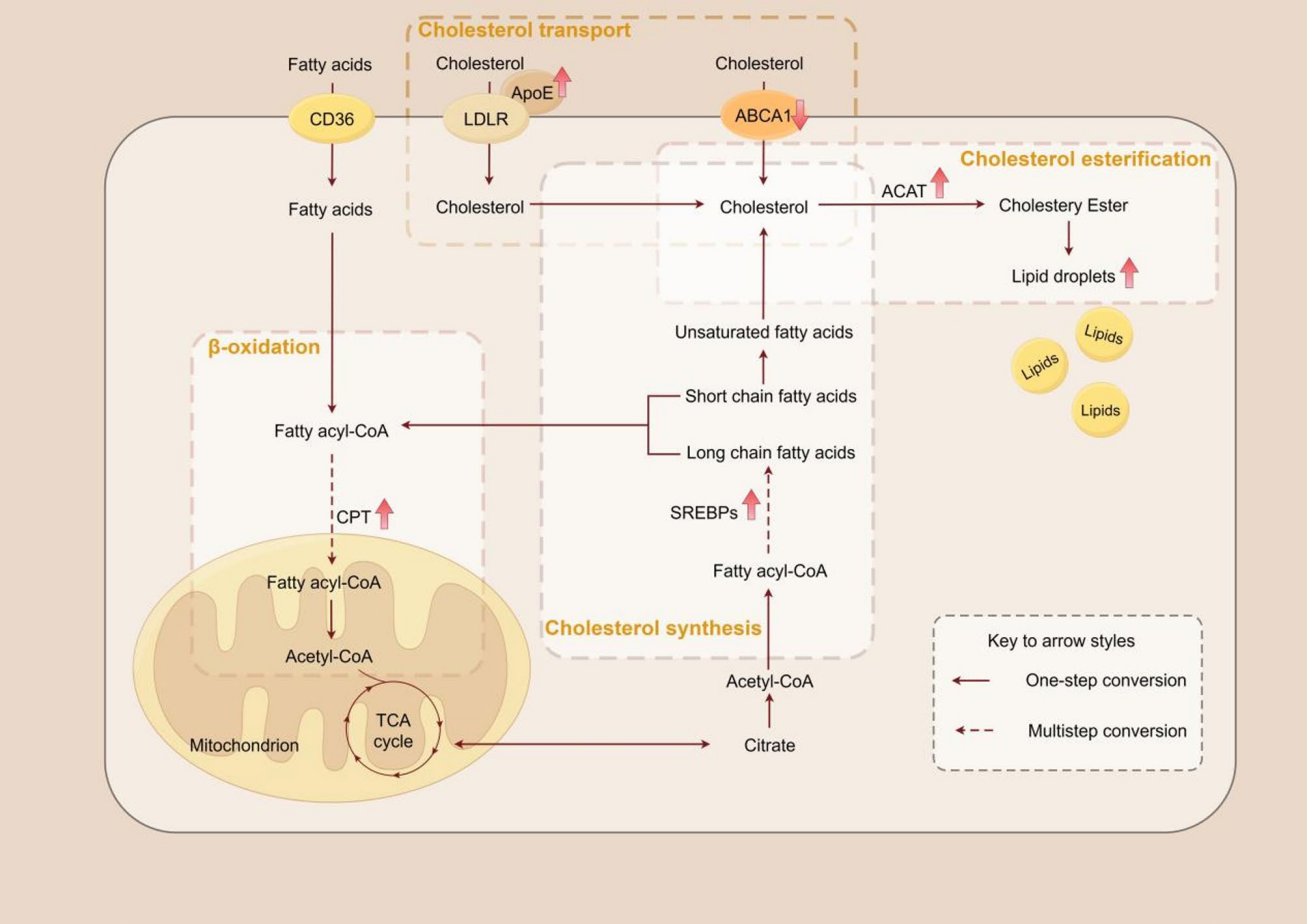
Lipid metabolism in Alzheimer’s Disease. Lipid metabolism in AD involves cholesterol synthesis, esterification and transport, and oxidation of fatty acids. The upward arrows indicate an increase in enzyme expression/activity and associated metabolic pathways. By targeting these enzymes and transporters, such as ACAT, ABCA1, CPT, and SREBPs, which regulate lipid metabolism and affect lipid synthesis, transport, and storage, it is possible to reduce AD pathology. This could potentially slow down or mitigate the progression of Alzheimer’s disease by modulating the abnormal lipid metabolism that is associated with the disease

Lipids are a diverse group of organic compounds widely found in living organisms, including cholesterol, fatty acids, phospholipids, and sphingolipids, among others. They play critical roles in cellular structure, energy storage, and signal transduction. The brain, being one of the most lipid-rich organs, relies on the transport and distribution of lipids to maintain homeostasis and neuronal function [92]. Cholesterol is essential for maintaining the fluidity and stability of neuronal cell membranes [93], and it also mediates neuroinflammation [94]. Fatty acids are key components of phospholipids, and the degradation of fatty acids in astrocyte mitochondria leads to neuroinflammation and neurodegeneration [95]. Phospholipids and sphingolipids are major components of biological membranes, crucial for maintaining the structural and functional integrity of cell membranes. These lipids not only provide a physical barrier but also give rise to metabolites involved in various cellular signaling processes [96]. In AD, lipid metabolism processes involve cholesterol synthesis, esterification, transport, and fatty acid oxidation, all of which are tightly regulated by a series of enzymes and transport proteins [97] (Fig. 2).

### Cholesterol metabolism in Alzheimer’s disease

Numerous studies have shown that elevated cholesterol levels are associated with an increased risk of dementia [98, 99]. A recent report by Livingston et al. identified elevated low-density lipoprotein cholesterol (LDL-C) levels as a risk factor for mid-life dementia, based on new evidence from large cohort studies involving over a million participants and a Mendelian meta-analysis of 27 studies [100, 101]. Paiva et al. [102] found that the downregulation of cholesterol biosynthesis genes in AD impairs memory function in hippocampal neurons under pathological conditions. Sterol regulatory element-binding proteins (SREBPs), including SREBP1 and SREBP2, are transcription factors involved in cholesterol and fatty acid synthesis. Spell et al. [103] found that polymorphisms in SREBP-1a affect the risk of AD in ApoE4 allele carriers. Overexpression of SREBP-2 in APP/PS1 mice results in Aβ deposition and tau aggregation typical of AD [104], and inhibiting SREBP-2 improves neurodegenerative pathology by significantly reducing Aβ production, abundance, and aggregation, as well as restoring synaptic function and alleviating cognitive and memory deficits [105]. Additionally, SREBP-2 is influenced by tau alterations in AD, causing the translocation of nuclear mSREBP-2, leading to disrupted cholesterol homeostasis in AD [106].

Cholesterol is stored in cells as cholesteryl esters (CE). Studies have shown that CE is an early upstream regulator of tau accumulation in iPSC-derived neurons from AD patients [107]. West et al. [108] similarly observed that inhibiting cholesterol esterification significantly increases Aβ-induced synaptic damage, and that using cholesterol ester hydrolase inhibitors can protect neurons. The biosynthesis of CE is catalyzed by Acyl-CoA acyltransferase (ACAT), which esterifies cholesterol to help cells reduce free cholesterol content and prevent excessive cholesterol accumulation in the membrane, maintaining cellular cholesterol balance. Studies have shown that ACAT inhibitors, such as CP-113,818, significantly reduce amyloid pathology in AD mouse models [109]. Another team used high concentrations of the ACAT1 inhibitor F12511 encapsulated in stealth liposomes to significantly improve amyloid pathology in aged 3xTg AD mice, reducing hyperphosphorylated tau and non-phosphorylated tau, as well as alleviating neuroinflammation [110]. Furthermore, AAV-mediated ACAT1 knockdown has potential therapeutic effects in AD, as delivery of AAV-ACAT1 to the brains of 10-month-old AD mice reduced brain Aβ and human amyloid precursor protein (hAPP) levels at 12 months of age [111].

The cholesterol synthesis capacity differs among various brain cell types. Cholesterol levels in the brain are regulated through the crosstalk between the plasma membrane and the endoplasmic reticulum, not only to meet the distinct cholesterol demands of different cells but also to regulate cellular functions [112, 113]. ApoE4, a key protein for cholesterol transport, is primarily synthesized and secreted by astrocytes in the brain. There are three ApoE isoforms (ApoE2, ApoE3, and ApoE4), with ApoE4 being the strongest genetic risk factor for sporadic Alzheimer’s disease (SAD). Recent studies show that by age 55, nearly all ApoE4 homozygotes exhibit AD pathology and higher levels of disease-related biomarkers. By age 65, over 95% of ApoE4 homozygotes show AD biological features in the brain or abnormal Aβ levels in cerebrospinal fluid, with 75% testing positive for amyloid scans [114]. Early detection of Alzheimer’s disease (AD) relies primarily on biomarkers in cerebrospinal fluid (CSF), such as Aβ42, Aβ42/Aβ40 ratio, p-Tau181, and total Tau (t-Tau). But the new study found that the ApoE4 gene affects the levels of these biomarkers, causing the same test to behave differently in different populations [115]. This means that if the ApoE4 status is not considered, it may result in misdiagnosis or missed diagnosis.ApoE4 variants not only increase the risk of AD but also influence the disease’s progression and severity [116]. ApoE4-containing microglia contribute to tau phosphorylation and neurotoxicity in an ApoE-dependent manner [117]. Shi et al. [118] showed that overexpression of low-density lipoprotein receptors reduces tau-related neurodegeneration associated with ApoE mechanisms.

Cholesterol reverse transport refers to the process of transporting cholesterol from peripheral tissues back to the liver through the bloodstream, where it is metabolized and excreted [119]. ATP-binding cassette transporter A1 (ABCA1) is a key protein in this reverse transport process and plays an essential role in the transport of cholesterol and phospholipids. It transfers excess cellular cholesterol and phospholipids to apolipoproteins, forming high-density lipoprotein (HDL)-like particles that maintain cellular cholesterol balance. In the brain, ABCA1 lipidates ApoE [120], facilitating Aβ clearance [121, 122], regulating peripheral vasculature and blood-brain barrier integrity, as well as anti-inflammatory signaling [123], influencing myelination, synaptogenesis, and neurotransmission [124], playing a crucial role in the onset and progression of AD. Holstege et al. [125] found a significant association between ABCA1 loss-of-function mutations and increased AD risk, based on exome sequencing data from 16,036 AD cases and 16,522 controls. Moulton et al. [126] found that ABCA1 peptide agonists promoted the formation of protective glial lipid droplets in ApoE4 humanized fly models.

### Fatty acid metabolism in Alzheimer’s disease

The brain heavily relies on astrocytes for oxidative phosphorylation (OxPhos) to degrade fatty acids (FAs) and maintain lipid homeostasis. Both the brain and blood in AD patients show alterations in fatty acid profiles, with notable differences in the lipidomic fatty acid profiles of CSF and plasma [127]. Specific changes in erythrocyte FA composition even occur prior to the onset of cognitive impairment [128]. Chen et al. [129] utilized metabolomics to identify polyunsaturated fatty acid (PUFA) metabolites in 3xTg mice and wild-type mice, finding that gut microbiota regulates AD pathology and cognitive dysfunction through PUFA-associated neuroinflammation.

Carnitine palmitoyltransferase (CPT), a multi-protein complex, is a rate-limiting enzyme in fatty acid oxidation. CPT1c, a neuronal subtype, is predominantly located in brain regions such as the hypothalamus, amygdala, and hippocampus, and plays a crucial role in neurodegenerative diseases, including AD [130]. Sarnowski et al. [131] identified a distinct association between CPT1A loci and insulin resistance in AD through multi-tissue epigenetic analyses. Upregulation of CPT1a expression in APP/PS1 mice enhances fatty acid oxidation in astrocytes, improving memory deficits [132].

### Lipid metabolism-related animal models of Alzheimer’s disease

Animal models have been instrumental in studying the link between lipid metabolism and AD. Through animal models related to lipid metabolism, researchers are able to better understand how lipid abnormalities affect nerve cell function, inflammatory response and proteostasis. These models provide valuable experimental evidence for developing therapeutic strategies against abnormal lipid metabolism and provide new research directions for early diagnosis and prevention of AD. The following in vivo models have been developed over the years (Table 2).

**Table 2:** Lipid metabolism-related animal models of Alzheimer’s disease

Model Type	Description	Intervention	Reference
High-fat diet-induced AD models	AD mice were given a high-fat diet to mimic the effects of disturbed lipid metabolism	High-fat diet for 5×FAD mouse model	[133]
		High-fat diet for Tg601 mice	[134]
		High-fat diet for 3xTg mice	[135]
Animal models of the altered lipid transport process	Targeting lipid transport	Deletion of the cholesterol sensor SCAP in C57BL/6 mice	[136]
		Transgenic knockin mouse expressing HaloTag-tagged ApoE	[137]
		Deletion of microglial ApoE4 in APP/PS1 mice	[138]
		Deletion of astrocytes ApoE in APP/PS1−21 mice	[139]
		ApoE4 knockin APP/PS1 mice	[140]
		Thy1-ApoE4/C/EBPβ double transgenic mouse model	[141]
		Neuronal specific Thy1-ApoE4/C/EBPβ double transgenic mice	[142]
		LDLR-targeted AD models	[118, 143]
		Pericyte LRP1-deficient in APP^Swe/0^mice	[144]
		LXR agonist GW3965 treatment for P301S/ApoE4 mice	[145]
		LXR agonist CE9A215 treatment for 3xTg-AD mice	[146]
		LXR agonists T1317 and GW3965 treatment for rats and C57Bl6/SJL respectively	[147]
Animal models of altered lipid metabolism processes	Targeting key enzymes for lipid metabolism	SREBP2 overexpression in APP/PS1 mice	[104, 148]
		Lovastatin (HMGCR inhibitor) treatment for rats	[149]
		CP−113,818 (ACAT inhibitor) for hAPP mice	[109]
		Avasimibe (ACAT inhibitor) treatment for Tg mice	[150]
		ACAT-knock out in Tg mice	[150]
		ACAT1 knockdown gene therapy in 3xTg mice	[111]
		Peptide agonist of ABCA1 treatment for humanized ApoE4 fly model	[126]
		NLAI (ABCA1-inducers) treatment for E3/4FAD mice	[120]
		Atorvastatin (Cholesterol inhibitor) treatment for Wistar rats injected Aβ1–42	[151]
		CPT1a-targeted APP/PS1 mice	[132]

## Immune-lipid metabolic interactions at the tissue level

### Immune-Lipid metabolic interactions in the choroid plexus

The choroid plexus (CP), located between the blood-brain barrier and CSF, consists of tightly connected epithelial cells, a vascular-rich stroma, and mesenchymal, glial, neuronal, and immune cells. It is the primary site of CSF production and also produces a variety of neurotrophic factors (NTFs) that circulate throughout different brain regions [152]. The CP serves as a selective and strictly regulated physiological gateway for immune cells entering the CNS, playing a key role in regulating solute exchange between the blood and CSF, mediating CNS immune surveillance and repair, and removing toxic waste, such as Aβ, to protect the brain.

CD4 + T helper (Th) cells in the CP stroma are essential for maintaining the expression of BDNF and leukocyte trafficking determinant factors. The CP’s immune environment controls its function, which, in turn, impacts CNS function and repair [153, 154]. The high secretory nature and strong molecular transport activity at the CP require high metabolic activity, which is linked to inflammation regulation. Lipid metabolic components may play important roles in modulating local immune responses. For instance, liver X receptors (LXRs) are critical for CP function and anatomical integrity, with LXR signaling regulating CSF fluid production, Aβ clearance, and inflammation modulation [155]. Systemic treatment with liver LXR agonists can enhance ApoE- and cholesterol-mediated Aβ transport to the CSF [147]. Endogenous LXR ligands, such as 24 S-hydroxycholesterol, induce ATP-binding cassette transporters ABCA1 and ABCG1 in an ApoE subtype-dependent manner, promoting cholesterol release from CP epithelial cells into the CSF [156]. Additionally, CP epithelial cells express the brain-specific cholesterol 24-hydroxylase CYP46A1, with its expression levels reduced in amyloid degeneration and aging mouse and human brains. Overexpression of CYP46A1 in amyloid-degenerative transgenic mice is associated with better cognitive performance and reduced brain inflammation [157]. Therefore, CP metabolism plays a crucial role in regulating inflammation, and lipid metabolism and immune homeostasis within the CP represent promising avenues for enhancing brain protection [158].

### Immune-lipid metabolic interactions in the blood-brain barrier

Vascular dysfunction is a major risk factor for neurological diseases, with early blood-brain barrier (BBB) disruption and/or dysfunction in AD often observed before the onset of dementia, neurodegeneration, or brain atrophy [159, 160]. The BBB consists of a plasma-brain cell barrier formed by the walls of cerebral capillaries and glial cells, as well as a plasma-CSF barrier formed by the choroid plexus, both of which block harmful substances from entering brain tissue. When the BBB is compromised, fibrinogen exudes into the CNS, where it is converted to fibrin after coagulation activation. Fibrin further activates glial cells and innate immune cells via CD11b/CD18 integrins, triggering pathological inflammation and oxidative damage, leading to neurodegenerative lesions.

The lipid nutrient metabolism and immune regulation of the BBB are highly interconnected processes. Excessive saturated fatty acids and monosaccharides in the diet may contribute to AD-related pathology by accelerating inflammation, metabolic inflammation, and systemic inflammation, driving BBB damage and neuroinflammation, which ultimately leads to synaptic dysfunction and memory and cognitive impairment [161]. High-density lipoprotein (HDL) reduces Aβ accumulation in the vasculature and dampens Aβ-induced endothelial inflammation, thus lowering AD risk [162–164]. Furthermore, BBB disruption is mediated by ApoE4. In ApoE4 mice, disrupted signaling in endothelial and pericyte cells earlier reflects progressive BBB failure, preceding postsynaptic damage and the development of behavioral defects 2–5 months later [165]. In terms of treatment, overcoming the BBB is a major challenge for AD therapies, as drugs must pass through the BBB to reach their target sites. Liposomes, with their unique phospholipid bilayer structure (similar to physiological membranes), are more compatible with the lipid layers of the BBB and facilitate drug entry into the brain, showing significant potential in AD therapy [166, 167].

### Immune-lipid metabolic interactions in the glymphatic system

The glymphatic system, a unique structure filled with CSF from small vascular perivascular spaces, facilitates the clearance of CNS-derived antigens, metabolites, and molecules (such as Aβ and tau), while regulating immune responses and fluid balance in the brain [168, 169]. In addition to waste removal, the lymphatic system functions as a distribution system for electrolytes, macromolecules, and other large compounds, providing an important route for the distribution of these molecules and improving the effectiveness of intrathecal drug delivery [170, 171].

The glial lymphatic system is thought to be mediated by aquaporin-4 (AQP4) expressed on astrocytes, which facilitates the exchange of CSF and interstitial fluid (ISF) within the perivascular spaces. This system primarily consists of the perivascular space surrounding arteries, AQP4 on astrocytic endfeet, and the perivenous space [172]. Physiological factors, including arterial pulsations, respiration, and CSF pressure gradients, drive the CSF flow from the subarachnoid space into deep brain structures via the perivascular space, and subsequently into the brain parenchymal spaces. The CSF metabolic products and toxic proteins are eventually drained through meningeal lymphatics to the cervical lymph nodes and peripheral lymphatic systems, maintaining CSF-ISF balance and clearing brain metabolites [173, 174].

Recent assessments of lymphatic function in aged versus young mice have shown that lymphatic function declines dramatically (by 80–90%) in older mice compared to younger controls [175]. In healthy young humans, CSF enters the brain parenchyma through the perivascular route, clearing solutes from the interstitial spaces and draining along the venous routes. In AD, compared to age-matched controls, patients frequently show abnormal enlargement of perivascular spaces, suggesting that vascular amyloidosis may reduce the influx of lymphatic CSF, with CSF stasis accelerating Aβ accumulation [176, 177].

Interestingly, reduced CSF-ISF exchange does not correlate with Aβ clearance, potentially due to the direct clearance of some Aβ via low-density lipoprotein receptor-related protein 1 (LRP1)-mediated trans-endothelial transport into the bloodstream. LRP1, a member of the LDL receptor family, plays a significant role in the endocytosis of tau proteins, and LRP1 gene silencing markedly inhibits tau internalization [143]. Additionally, LRP1 promotes mitochondrial transfer from astrocytes to neurons, reducing neuronal sensitivity to acute stress [178]. Neuronal LRP1 deletion leads to neuroinflammation [179]. Thus, LRP1 may serve as a bridge between lipid metabolism and immune regulation in AD progression. Moreover, dietary supplementation with polyunsaturated fatty acids (PUFAs) can improve glial lymphatic function, reduce neuroinflammation and cerebrovascular dysfunction, and ultimately improve cognitive abilities [180, 181].

## Immune-metabolic interactions at the cellular level

### Membrane-associated signal transduction

Extracellular vesicles (EVs) are membrane-bound vesicles released into the extracellular space, carrying proteins, miRNAs, and metabolites into the circulatory system. Their role in intercellular communication has garnered increasing attention and is believed to be involved in the pathogenesis of neurodegenerative diseases, including AD [182–184]. In AD, brain-derived EVs not only mediate synaptic dysfunction but also propagate tau protein in hippocampal GABAergic interneurons, ultimately leading to neuronal dysfunction [185]. When these EVs are released from the brain into the periphery, they provide AD-specific immune-metabolic biomarkers [186–188]. For example, the inflammatory factor ITGB1 is significantly elevated in astrocyte-specific EVs enriched with brain-derived AD EVs, and this elevation correlates with brain Aβ and tau burden in independent cohorts [189]. Compared to normal controls, brain-derived EVs in AD significantly alter glycerophospholipid and sphingolipid levels, particularly increasing alkenylglycerophosphoethanolamine and decreasing polyunsaturated acyl-lipid content. Changes were also observed in the acyl-chain content of amide-linked sphingolipids and ceramides. The most notable change was a two-fold reduction in lipids containing the anti-inflammatory/pro-resolving eicosapentaenoic acid [190]. Recently, a research team has developed a method for treating AD using mesenchymal stem cell-derived extracellular vesicles (MSC-EVs-SHP2) with high expression of tyrosine phosphatase-2 (SHP2). In AD mouse models, MSC-EVs-SHP2 were shown to significantly induce mitochondrial engulfment in neuronal cells, thereby alleviating mitochondrial damage-mediated apoptosis and NLRP3 inflammasome-induced neuroinflammation, ultimately preventing synaptic loss and cognitive decline [191]. Therefore, EV-mediated immune-metabolic responses provide a promising new platform for AD treatment.

ApoE, the main cholesterol transporter in the brain, mediates the transfer of cholesterol and other lipids between neurons and glial cells. ApoE achieves lipid transport by interacting with members of the LDL receptor family on the surfaces of neurons and glial cells. After binding to these receptors, ApoE releases lipids into the cells, providing the material basis for their physiological functions. Studies have shown that pro-inflammatory stimuli can induce metabolic reprogramming in microglial cells. In induced pluripotent stem cell-derived microglia-like cells (iMGLs), ApoE4 iMGLs showed a significant increase in lipid droplet content and a reduction in genes related to lipid degradation and membrane fatty acid transporters, such as CD36 [192, 193]. ApoE4-induced lipid accumulation increases the inward rectifier potassium (Kir) current in the neuronal cell membrane, hyperpolarizing the resting membrane potential and reducing neuronal excitability [194]. Transcriptional profiling revealed that ApoE4 iMGLs are significantly enriched in HIF-1, JAK-STAT signaling, and cytokine-cytokine receptor interactions, suggesting a strong pro-inflammatory response [195].

**Fig. 3:**
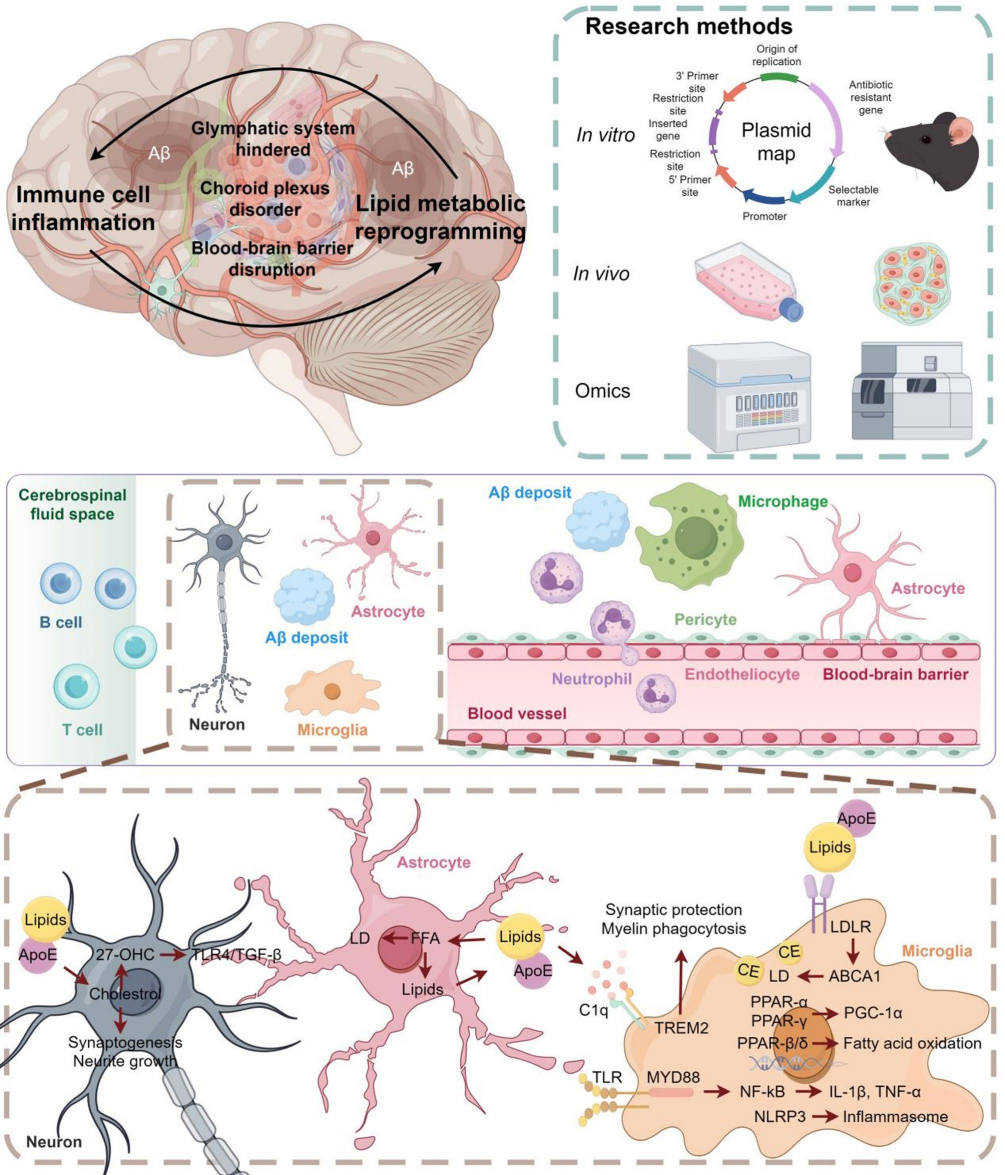
Immunometabolism in Alzheimer’s disease. The diagram illustrates the interaction between the immune response and lipid metabolism in AD, covering complex mechanisms from the tissue level to the cellular level. The figure presents the immune system activation, inflammatory response, and how lipid metabolism affects the healthy state of nerve cells, revealing the pathological process and potential therapeutic targets in AD. Current methodologies used to study the interaction between immunity and lipid metabolism primarily include in vivo and in vitro experiments, along with bioinformatics approaches such as omics analyses. In vivo studies commonly employ transgenic or gene knockout mouse models, LPS-induced neuroinflammation models, and high-fat diet-induced models. In vitro experiments typically utilize central nervous system immune cells to investigate the crosstalk between lipid metabolism and immune responses. Omics approaches such as RNA sequencing, lipidomics, and single-cell sequencing, facilitate the identification of key regulatory networks involving inflammatory factors and lipid metabolism-related genes. Future research on the interaction between immunity and lipid metabolism in AD should focus on identifying critical molecules and signaling pathways, ultimately facilitating the construction of a more comprehensive immune–lipid metabolism interaction network in AD

Triggering receptor expressed on myeloid cells 2 (TREM2) is a transmembrane immune receptor predominantly expressed on microglial cells in the brain and macrophages in the periphery. Loss-of-function (LOF) variants of TREM2 increase the risk of AD [196, 197]. In mice and humans carrying TREM2 LOF variants, higher levels of apoptotic synapses have been observed [198]. In AD, TREM2 plays a crucial regulatory role in both central and peripheral immune and metabolic responses, making it a potential target for AD immunometabolic therapy [199–202]. In the CNS, TREM2 serves as a surface receptor required for microglial response to neurodegeneration. It supports microglial energy and biosynthesis metabolism, making their activation possible during AD [203, 204], while also reducing Aβ seeding and inhibiting disease-associated microglial activation [205]. However, Jain et al. [206] reported that prolonged use of activated TREM2 antibodies might exacerbate Aβ-induced tau pathology, highlighting the importance of considering the duration and dosage in clinical TREM2 interventions. Additionally, Aβ-induced microglial depolarization, K + inward current induction, cytokine expression and secretion, migration, proliferation, apoptosis, and morphological changes are all dependent on TREM2 [207–209]. TREM2 also protects complement-mediated synapse loss through its interaction with complement C1q during neurodegeneration [210]. TREM2 mediates lipid patterns associated with neurodegeneration [211] and promotes myelin engulfment, regulating cholesterol metabolism in microglia under chronic phagocytic stress. Its LOF leads to pathogenic lipid accumulation in microglia [212, 213]. Replacing TREM2-deficient microglia with TREM2 wild-type cells derived from hematopoietic cells has been shown to correct microglial dysfunction in the 5×FAD mouse model, offering a potential new treatment approach for AD [91]. In the periphery, TREM2 affects lipid metabolism by regulating the onset and progression of obesity and its complications, including hypercholesterolemia, atherosclerosis, and non-alcoholic fatty liver disease. All of these altered lipid metabolic processes can influence AD progression through their effects on inflammation, insulin resistance, and AD pathology [214] (Table 3; Fig. 3).

**Table 3:** AD risk genes associated with both the lipid metabolism and the immune system

Gene symbol	Lipid metabolic role	Immune function	AD-related pathology
ApoE4	Major apolipoprotein in the brain; transports cholesterol and phospholipids [192]	Modulates inflammation and microglial activation [137, 221]	Strongest genetic risk factor for late-onset AD; affects Aβ clearance and neuroinflammation [114]
TREM2	Senses lipid debris; regulates lipid uptake in microglia [211]	Controls microglial phagocytosis and anti-inflammatory state [91]	TREM2 variants (e.g. R47H) impair Aβ clearance and increase AD risk [197]
ABCA1	Promotes cholesterol efflux and ApoE lipidation [120]	Indirectly regulates microglial response via ApoE lipidation [156]	Impaired ABCA1 function linked to reduced ApoE function and increased Aβ deposition [121]
NLRP3	Activated by cholesterol crystals and fatty acids [191]	Key component of inflammasome activation [34]	Drives chronic neuroinflammation in AD; inflammasome activation worsens Aβ pathology [34]
CPT1A	Controls fatty acid β-oxidation, mitochondrial lipid metabolism [130]	Influences immune cell energy balance [132]	Altered energy metabolism may contribute to astrocytes dysfunction in AD [132]
LRP1	Mediates ApoE and Aβ transport; involved in lipoprotein clearance [144]	Regulates cellular signaling and immune neuroinflammation in the brain [179]	Mediates tau internalization [143]
TLR4	Can be activated by oxidized lipids and fatty acid derivatives [225]	Recognizes danger signals, triggers innate immune response [226]	TLR4 activation leads to neuroinflammation and is linked to cognitive impairment [77]
PPARs	Regulates mitochondrial biogenesis and fatty acid oxidation [234]	Suppresses inflammation, promotes microglial phagocytosis [133]	PPAR-α agonists reduce amyloid pathology and reverse memory deficits [233]

### Intracellular signaling pathways

ApoE4 not only facilitates lipid transport at the cell membrane but also plays a differential role in amyloid and tau pathology, lipid metabolic changes, glial cell reactivity, neurodegeneration, demyelination, and BBB dysfunction in a time-space interactive manner. ApoE4 expressed in various types of CNS cells (including astrocytes, neurons, microglia, oligodendrocytes, and endothelial cells) has distinct roles in AD pathogenesis [215]. Lee et al. [216] employed RNA-seq, metabolomics, spatial transcriptomics, and mass spectrometry imaging to report that ApoE4 exacerbates amyloid plaque-induced microglial activation and lipid metabolic changes, emphasizing the central role of ApoE4 in regulating microglial immune metabolism. In AD, ApoE4 is linked to lipid droplet damage in microglia [217], and lipid-laden microglia carrying APOE4 increase tau phosphorylation and neurotoxicity [218]. ApoE4 microglia derived from AD patient-induced pluripotent stem cells and ApoE4-associated tauopathy mouse models exhibit significantly increased cholesterol biosynthesis and accumulation, which correlates with persistent microglial activation and elevated antigen presentation via the major histocompatibility complex II, followed by T-cell infiltration [219]. ApoE4 expression downregulates complement and lysosomal pathways and promotes stress-related responses [220]. Moreover, APOE4 microglia show altered morphology, slower movement toward Aβ, and reduced surveillance and phagocytosis of Aβ [221, 222]. In astrocytes, APOE4 induces lysosomal cholesterol chelation, reducing free cytoplasmic cholesterol, thereby leading to lipid dysregulation by increasing cholesterol biosynthesis and reducing efflux [192]. Selective removal of ApoE4 from astrocytes confers strong protection against tau-mediated neurodegeneration and reduces microglial synaptic phagocytosis [223]. In oligodendrocytes, studies by Manolis Kellis and Li-Huei Tsai have demonstrated that ApoE4 alters cholesterol biosynthesis, transport, and localization in both human and mouse oligodendrocytes, correlating with reduced myelination and endoplasmic reticulum stress. Pharmacological enhancement of cholesterol transport can increase myelination in aged ApoE4 mice and improve their learning and memory abilities [224].

Additionally, lipid peroxidation products can serve as endogenous danger signals, binding to toll-like receptors (TLRs) on microglial and peripheral immune cells, activating the TLR4/NF-κB pathway, and inducing early-stage pro-inflammatory M1 microglial-mediated Aβ phagocytosis defects, promoting the release of pro-inflammatory factors that damage neurons and accelerate AD progression [225]. 25-hydroxycholesterol (25-HC) promotes the production of brain cytokine IL-1β and leukocyte infiltration in LPS-induced neuroinflammation models [73]. 27-hydroxycholesterol (27-OHC) causes inflammatory neuronal damage by activating the TGF-β/NF-κB signaling pathway and induces inflammatory damage in astrocytes via the TLR4/TGF-β signaling pathway, leading to the release of inflammatory cytokines [226]. Therefore, cholesterol depletion can also serve as a biomarker for chronic neuroinflammation.

### Regulation at the nuclear transcription level

The peroxisome proliferator-activated receptor (PPAR) family is a key nuclear receptor involved in lipid metabolism, consisting of three subtypes: PPAR-α, PPAR-β/δ, and PPAR-γ, all of which have a negative feedback relationship with inflammation. PPARs regulate cholesterol transport to the mitochondria and lipid metabolism in neurons. Recently, due to their additional anti-inflammatory and neuroprotective effects, they have garnered attention as potential therapeutic targets for neurodegenerative diseases [227, 228]. PPAR-α and PPAR-β/δ primarily stimulate oxidative lipid metabolism, whereas PPAR-γ is involved in lipid cell assimilation through anabolic pathways [229]. In AD brain tissue, gene expression of PPAR-α and PPAR-γ coactivator-1α (PGC-1α) is significantly reduced [230]. PPAR-γ agonists enhance Aβ phagocytosis and reduce the inflammatory cytokine IL-1β [231]. Targeting PPAR-γ can suppress the pro-inflammatory M1 phenotype in microglia, promote microglial phagocytosis, inhibit neuroinflammation, and improve cognitive deficits caused by a high-fat diet in the 5×FAD mouse model [133]. In APP-PSEN1 ΔE9 mice, PPAR-α agonists reduce amyloid pathology and reverse memory deficits, anxiety symptoms, and cognitive decline [232]. The exposure to inflammatory factor IL-15 promotes PPAR-β/δ pathway activation, suppresses aerobic glycolysis, and enhances oxidative metabolism and fatty acid oxidation, thus counteracting metabolic stress-induced CD8 + T-cell apoptosis [233]. Furthermore, PPARs can regulate lipid deposition in vascular wall cells, reducing the risk of atherosclerosis [234], which is significant for AD prevention, as cerebrovascular pathology is closely linked to AD development.

## Summary and prospect

Immune cells such as microglia can recognize and clear abnormal substances such as Aβ in the early stage of AD, while excessive immune responses can cause chronic neuroinflammation and accelerate neuronal damage and AD disease process. Lipid metabolism is able to affect the structure and function of the cell membrane, provide energy to the brain, and participate in cell signaling. Abnormalities in these processes are closely related to the disease progression of AD. The intersection between lipid metabolism and immune response, such as ApoE4, TREM2, and PPAR pathway, further amplified the disease progression of AD. However, many key questions remain unresolved, such as which other lipid metabolic enzymes, receptors or lipid metabolite supplements can regulate immune cell function and can be used to prevent, alleviate, or treat AD; whether immune cells can be divided into different metabolic subgroups according to their different lipid metabolic states and have specific functions of anti-neuroinflammation; how spatial and temporal changes of immune factors and metabolic factors in the progression of AD, how they form a positive feedback pathway in the progress of AD, and how to target lipid-immune interactions to intervene in the process of AD still need further study. In order to answer these questions, in-depth mechanistic research can be conducted through gene-editing and genetic breeding, which will help to more completely reveal the complex role network of lipid metabolism and immune response in AD, and provide new ideas for revealing the pathogenesis of AD.

## Data Availability

No datasets were generated or analysed during the current study.
